# Proteomic analysis of the postsynaptic density implicates synaptic function and energy pathways in bipolar disorder

**DOI:** 10.1038/tp.2016.224

**Published:** 2016-11-29

**Authors:** M Föcking, P Dicker, L M Lopez, M Hryniewiecka, K Wynne, J A English, G Cagney, D R Cotter

**Affiliations:** 1 Department of Psychiatry, Royal College of Surgeons in Ireland, Education and Research Centre, Beaumont Hospital, Dublin, Ireland; 2 Departments of Epidemiology and Public Health, Royal College of Surgeons in Ireland, Dublin, Ireland; 3 Proteome Research Centre, UCD Conway Institute of Biomolecular and Biomedical Research, School of Medicine and Medical Sciences, University College Dublin, Dublin, Ireland; 4 Department of Psychiatry, Beaumont Hospital, Dublin, Ireland

## Abstract

The postsynaptic density (PSD) contains a complex set of proteins of known relevance to neuropsychiatric disorders such as schizophrenia and bipolar disorder. We enriched for this anatomical structure in the anterior cingulate cortex of 16 bipolar disorder samples and 20 controls from the Stanley Medical Research Institute. Unbiased shotgun proteomics incorporating label-free quantitation was used to identify differentially expressed proteins. Quantitative investigation of the PSD identified 2033 proteins, among which 288 were found to be differentially expressed. Validation of expression changes of DNM1, DTNA, NDUFV2, SEPT11 and SSBP was performed by western blotting. Bioinformatics analysis of the differentially expressed proteins implicated metabolic pathways including mitochondrial function, the tricarboxylic acid cycle, oxidative phosphorylation, protein translation and calcium signaling. The data implicate PSD-associated proteins, and specifically mitochondrial function in bipolar disorder. They relate synaptic function in bipolar disorder and the energy pathways that underpin it. Overall, our findings add to a growing literature linking the PSD and mitochondrial function in psychiatric disorders generally, and suggest that mitochondrial function associated with the PSD is particularly important in bipolar disorder.

## Introduction

The postsynaptic density (PSD) is a highly organized structure attached to the postsynaptic neuronal terminal. It comprises a complex network of cytoskeletal scaffolding and signaling proteins that facilitate the movement of receptor and signaling complexes. The PSD is critical for normal neurotransmission and synaptic plasticity^1^ through the modulation of signaling mechanisms involving n-methyl-d-aspartate, α-amino-3-hydroxy-5-methyl-4-isoxazolepropionic acid and metabotropic glutamate receptors. Synaptic plasticity is implicated in neuropsychiatric disorders,^2, 3, 4, 5^ and thus the PSD is implicated.^6, 7, 8, 9, 10, 11, 12, 13^ However, although known constituents of the PSD have been implicated in bipolar disorder and schizophrenia at both gene^14, 15, 16^ and protein expression levels,^14, 17, 18, 19, 20^ no major findings are reported to contribute to bipolar disorder risk.^21, 22^ As synaptic plasticity is highly dependent on mitochondrial function,^23^ energy metabolism acting at the level of the PSD may underpin PSD dysfunction in bipolar disorder and indeed other neuropsychiatric disorders.

Mass spectrometry-based proteomic methods have the ability to reliably identify and quantify several thousands of disease-associated protein changes derived from complex anatomical structures. The reliable quantitation of low-abundance proteins within specific cellular compartments until recently has been challenging and this has led to a shift in the use of pre-fractionation enrichment methods combined with mass spectrometry-based proteomic techniques. This approach has successfully yielded a detailed characterization of the PSD proteome in rodents and in healthy postmortem human brain tissue.^24^ The differential expression of the PSD in schizophrenia compared with controls was first reported recently by our team^25^ highlighting altered pathways of endocytosis, long-term potentiation and calcium signaling in schizophrenia. The identified PSD proteome (including a gene critical to synaptic plasticity—*MAPK3*) was significantly associated in gene set enrichment analysis with schizophrenia, validating independent reports of PSD enrichment in schizophrenia. Furthermore, numerous mitochondrial proteins were differentially expressed in the PSD schizophrenia proteome and these changes were not associated with antipsychotic administration.^25^ This was in keeping with previous studies of the brain in neuropsychiatric diseases.^26, 27, 28, 29, 30, 31, 32, 33, 34, 35, 36^ These findings are of relevance to bipolar disorder, considering the known overlap with schizophrenia in terms of clinical presentation, genomic, structural imaging,^37^ transcriptome and protein expression.^26^ To date, no study has assessed the protein expression of the PSD in the cortex in bipolar disorder compared with controls.

In the current investigations, we enriched for the PSD in the anterior cingulate cortex (ACC) in bipolar disorder and in control human brain samples. This is a candidate brain region in both bipolar disorder and schizophrenia.^38, 39, 40, 41^ We undertook a label-free liquid chromatography–mass spectrometry (LC-MS/MS) investigation to identify disease-associated changes in protein expression within the PSD in bipolar disorder compared with controls. We hypothesized three findings. First, that the altered protein expression would overlap with that observed in schizophrenia PSD.^25^ Second, that a distinct pattern of altered protein expression would emerge in keeping with genomic and mRNA expression bipolar disorder studies. Third, that mitochondria-associated proteins would be differentially expressed in this PSD-enriched fraction in bipolar disorder (based on our own and other recent studies implicating mitochondrial function in major psychiatric disorders).

## Materials and methods

### Samples

Human postmortem brain tissue of the supragenual (BA24) ACC was obtained from the Stanley Medical Research Institute's Array Collection (www.stanleyresearch.org). The series consists of 105 subjects, including 35 schizophrenia, 35 bipolar disorder and 35 control cases. Information on prescribed psychotropic medication is provided by the Stanley Medical Research Institute.

A subset of 20 bipolar disorder samples and 20 control samples were selected from the series to match as closely as possible for age and tissue pH.^35, 42^ Table 1 provides detailed demographic information on these subjects. To obtain enough tissue for the enrichment protocol, two samples were subpooled based on the Euclidean distance (Supplementary Table 1). Investigators were blind to group identity until completion of the data analysis. Ethical approval (Application No. REC080) was granted by the Royal College of Surgeons in Ireland Research Ethics Committee. Two subpooled samples were subsequently excluded from the bipolar disorder group as we were unable to obtain enough PSD protein for our analyses.

### Comparing the PSD in ACC in bipolar disorder and controls

The enrichment for the PSD was undertaken using methods established previously and recently validated for postmortem brain material.^24^ In brief, the method involves differential sucrose centrifugation and further fractionations by Triton X-100 extraction first at pH 6, and then at pH 8, leading to the separation of the PSD fraction (for details see Supplementary Methods and our previous work^25^).

### Sample preparation for mass spectrometry

Fifty micrograms of protein from each homogenate was denatured in 10 μl 2% RapiGest solution (Waters, Elstree, UK) at 80°C for 10 min. Samples were subsequently reduced in the presence of 50 mM TCEP (tris2-carboxyethylphosphine) (Sigma Aldrich, Wicklow, Ireland) at 60°C for 60 minutes and alkylated in the dark with 200 mM iodoacetic acid acid (Sigma Aldrich). Protein was digested with 5 μg of sequence grade modified trypsin (Promega, Southampton, UK), in a 37°C shaking incubator for 16 hours. Digestion was stopped and the RapiGest precipitated with formic acid (0.1% v/v). After digestion, peptides were resuspended in 0.5% trifluoroacetic acid, dried in an Eppendorf Vacufuge (Eppendorf, Hauppauge, NY, USA) and desalted using ZipTips (Millipore, Bedford, MA, USA).

### Mass spectrometry analysis

Label free LC-MS was performed on a Thermo Scientific Q Exactive mass spectrometer connected to a Dionex Ultimate 3000 (RSLCnano) chromatography system. Tryptic peptides were resuspended in 0.1% formic acid. Each sample was loaded onto a fused silica emitter (75 μm ID, pulled using a laser puller (Sutter Instruments P2000)), packed with Reprocil Pur C18 (1.9 μm) reverse phase media and was separated by an increasing acetonitrile gradient over 120 minutes at a flow rate of 250 nL/min. The mass spectrometer was operated in positive ion mode with a capillary temperature of 320°C, and with a potential of 2300V applied to the frit. All data was acquired with the mass spectrometer operating in automatic data dependent switching mode. A high resolution (70,000) MS scan (300–1600 m/z) was performed using the Q Exactive to select the 12 most intense ions prior to MS/MS analysis using HCD.

### Data processing and analysis

We collected two types of mass spectrometry data in the experiments. It is formally a ‘shotgun' experiment and the collected tandem mass spectra were used to identify the proteins. The parent ion signal (MS1 scan) on high resolution instruments such as the Q Exactive used is widely used to calculate protein abundance in so-called ‘label-free' proteomics experiments.^43^ The signal for each peptide derived from a given protein is integrated over time (the time it takes to enter the instrument via HPLC), and the summed signals are used to estimate the relative abundance of each protein. To analyze the data, we used the MaxQuant programme specifically for label-free experiments using high resolution instruments supported by Andromeda as a database search engine for peptide identification.^44^ This program has several statistical control steps to ensure that only high quality reliable ion signals are accepted.

Label free quantitation (LFQ) was performed as previously described.^45^ Carbamidomethylation was defined as a fixed modification, while oxidation and acetylation of the protein N-terminus were defined as variable modifications. Only peptides with seven or more amino acid residues were allowed for identification. Additionally, at least one unique peptide was required to identify a protein. The cut off for false discovery rate for peptide and protein identification was set to 0.01. The label free algorithm takes the maximum number of identified peptides between any two samples and compares the intensity of these peptides to determine peptide ratios.

LFQ intensity values were used for protein quantification across the groups.

Raw LFQ intensities were extracted from the MaxQuant software and log base 2 transformed prior to analysis to eliminate distributional skew and to give approximate normality. To avoid bias associated with protein under-representation between groups, proteins were excluded in cases where there was less than 50% availability of the LFQ intensities in each biological group. After data filtering, 45872 LFQ values remained. Regression normalization was performed to remove technical variation between samples.^46^

### Statistical analysis

Protein intensities were screened for anomalous and missing data before log transformation (log base 2) and quantile regression normalization. A two-sample *t*-test (two-sided) analysis was performed on each protein comparing bipolar disorder with controls.

Significance testing was performed at the 5% level, and a false discovery rate (FDR)^47^ of 5% was used to flag those protein identifications deemed statistically significant after adjustment for multiple comparisons. Fold changes were obtained from an exponentiation (power of 2) of the difference in group means. Fold changes <1 were inverted (−1/fold change) to present a consistent scale of measurement.

Family-wise analysis was performed for two families of proteins. Mitochondrial dysfunction and the septin family were investigated as they were highlighted from pathway analysis and visual inspection, respectively. A family-wise *P*-value for the difference between groups was obtained from a nested model (analysis of variance) of the corresponding proteins (*n*=39 mitochondrial dysfunction, *n*=9 septins).

The management of data and statistical analyses were carried out with the SAS version 9.2 statistical software (SAS Institute, Cary, NC, USA) and R version 3.1 statistical software (R Foundation for Statistical Computing, Vienna, Austria).

### Classification of findings

See Supplementary Methods for methods of classification of protein changes using DAVID NIH, Search Tool for the Retrieval of Interacting Genes and Ingenuity Pathway Analysis.

### Validation of differentially expressed proteins

Proteins were selected for validation based on their degree of differential expression and membership of the main processes and protein families implicated by our current proteomic analysis and comparison with gene expression literature,^29, 48^ namely mitochondrial function (NDUFV2 and SSBP), endocytosis (DNM1 and DTNA) and a member of the Septin family (SEPT11). Validation was undertaken using antibodies to these proteins in the same above-mentioned bipolar disorder and control samples from the Stanley Foundation Array series and animal model of haloperidol-treated rats. For further details on the procedure and specificity of the antibodies used for this study see Supplementary Methods.

## Results

### Identification of PSD proteins and pathways dysregulated in schizophrenia

Thirty-six subjects pooled into 18 samples (8 pairs bipolar disorder and 10 pairs of control) were studied (Table 1). A total of 2867 proteins were identified by tandem mass spectrometry (Q-Exactive) after data input to the MaxQuant bioinformatics software (Max-Planck Institute for Biochemistry, Munich, Germany). Proteins were excluded if they were detected in less than 50% of samples from each group, leaving 2033 proteins for inclusion in the final statistical analysis (Supplementary Table 2).

Six hundred twenty proteins were differentially expressed in bipolar disorder compared with controls (*P*⩽0.05). Two hundred eighty-eight proteins were found to be significantly differentially expressed after correcting for multiple testing with FDR<0.05 (Supplementary Table 2 and Supplementary Figure 1).

**Figure 1:**
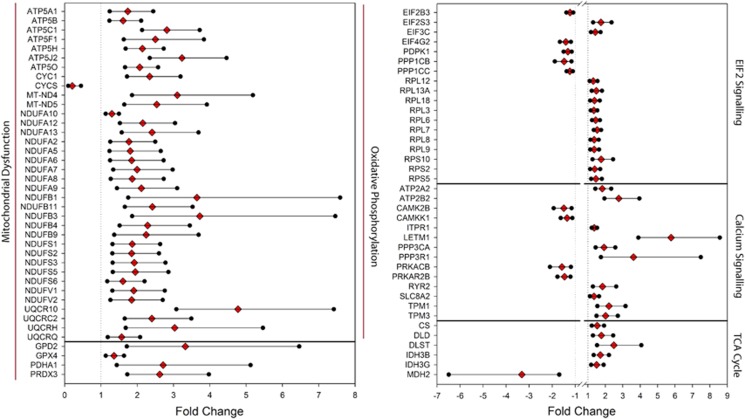
Forest plots of pathway analysis findings. The top five significant canonical pathways identified among the false discovery rate (FDR)-positive 288 differentially expressed proteins between bipolar disorder and control samples with fold changes and 95% confidence intervals are presented.

We asked whether the 288 PSD proteins contained functionally related protein subsets. Five pathways were found to be significantly enriched when analyzed by Ingenuity Pathway Analysis: oxidative phosphorylation, mitochondrial dysfunction, EIF2 signaling, calcium signaling and the TCA cycle (Table 2 and Figure 1).

Furthermore, both the septin and mitochondrial dysfunction set of proteins were differentially expressed in a family-wise analysis (both *P*-values <0.0001). The overall fold change for the nine septins in the data set was 1.60, with a 95% confidence interval from 1.34 to 1.92. The 39 mitochondrial dysfunction proteins showed an overall fold change of 2.05 (95% confidence interval 1.57–2.67).

### Confirmation of proteomic findings

#### Validation using human samples

**Figure 2:**
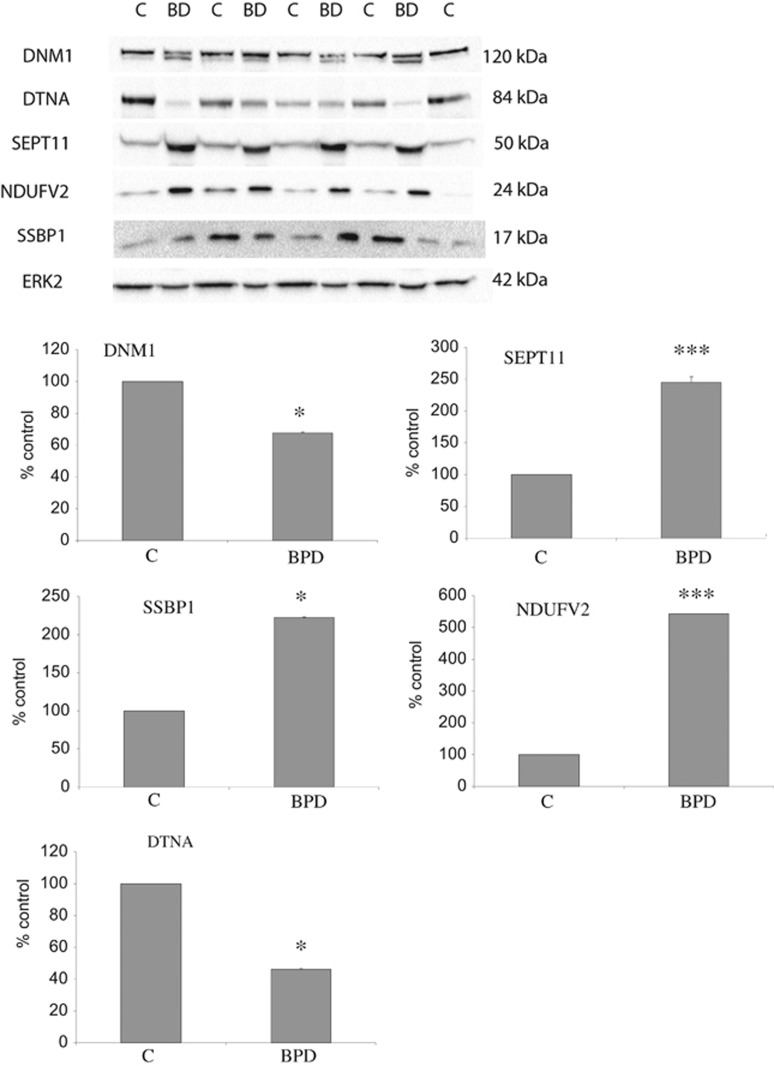
Validation of differentially expressed proteins. Protein expression changes were determined in the 18 subpooled cases of the Stanley Medical Research Institute Array Collection using western blot analysis. Representative images and the means of three independent experiments are presented. Error bars indicate s.d. Western blots were prepared using lysates of subpools of anterior cingulate cortex samples from patients with bipolar disorder (BD or BPD) and control subjects (C). Immunoblots were incubated with antibodies that specifically recognize Dynamin (DNM1) at 120 kDa, Dystrobrevin (DTNA) at 84 kDa, Septin 11 (SEPT11) at 50 kDa, (NDUFV2) at 24 kDa, single-stranded DNA-binding protein (SSBP) at 17 kDa and ERK2, used as a loading control, at 42 kDa. The images show a typical blot and the corresponding graphs represent the signal intensity of the designated antibody measured by densitometry and corrected by the signal intensity of ERK2. The mean of three independent experiments is presented. Error bars indicate s.d. ERK2 showed no significant differences between disease and control (*P*=0.66). *P<0.05, ***P<0.001. In keeping with our liquid chromatography–mass spectrometry (LC-MS/MS) experiments, DNM1 and DTNA1 expression levels were reduced and SEPT11 and NDUF2 were found to be increased. For DNM1, bipolar disorder cases showed two bands, indicating potential post-translational modifications of the protein. However, we measured the band at 120 kDa for consistency. SSBP was found to be decreased in the MS data but increased by western blotting. This most probably is due to the antibody recognizing different/all isoforms of the protein.

Validation was undertaken for proteins from different classes, namely mitochondrial function, endocytosis and septin family using western blotting for DNM1, DTNA, NDUFV2, SSBP1 and SEPT11 in the PSD-enriched samples from the Stanley Foundation Array series. See Figure 2 and Table 3 for the details of this validation work.

#### Validation using Haloperidol-treated rats

We previously explored the proteomic effect of antipsychotic medication (haloperidol) on the PSD using a rat model of antipsychotic drug treatment.^25^ Of the 26 proteins in the treatment group found to be statistically differentially expressed compared with controls, four were included in our list of significantly differentially expressed protein in the bipolar disorder PSD (*P*<0.05; FAM162A, MCCC1, RIN1 and mTOR), and one in our list of 288 FDR-corrected proteins in the bipolar disorder PSD (FAM162A), and also altered in the same direction. These findings suggest that differential expression of these proteins may be drug-related. However, new western blot analyses for the findings in this study demonstrated that DNM1, DTNA, NDUFV2, SEPT11 and SSBP1 were not significantly altered in rats treated with haloperidol (Table 3), indicating that the differential expression of these latter proteins in bipolar disorder PSD is not antipsychotic treatment-related. Overall, as the majority of expression changes observed by us in human samples were not seen in the rat antipsychotic model, a purely pharmacological explanation for the majority findings seems less likely (albeit using a different species, and a single antipsychotic compound).

### Comparison with schizophrenia PSD findings

We compared the above findings with the previous data obtained from the schizophrenia PSD samples^25^ (LTQ ORBITRAP, see Supplementary Tables 3 and 4 for details of overlapping proteins and pathways). Taking all 143 differentially expressed schizophrenia PSD proteins into consideration (*P*<0.05), we found an overlap of 54.5% with the current bipolar PSD differentially expressed data set (78/143), indicating a shared pathophysiology in keeping with past genetic and proteomic^49^ investigations.

### Mitochondrial findings using MitoCarta

As proteins linked to ‘mitochondrial dysfunction' were prominent in the list of differentially expressed proteins in the current study, we asked whether the set of 288 proteins were more likely to have a mitochondrial location in the cell.

Using MitoCarta (www.broadinstitute.org/pubs/MitoCarta/)^50^ we found 97 of the 288 differentially expressed proteins (FDR) as having a mitochondrial localization (Supplementary Table 5).

There was an over-representation of mitochondrial proteins differentially expressed within the PSD proteome of bipolar disorder compared with controls (according to MitoCarta, *P*<0.01 Fisher's Exact test). As a group, mitochondrial proteins were generally upregulated (Figure 1 and Supplementary Table 5 (83/97)). Overexpression of the mitochondrial-associated protein NDUFV2 was confirmed by western blotting (see validation work).

## Discussion

In this work, we used proteomic methods to study the PSD in bipolar disorder. We provide robust data implicating this multiprotein complex in bipolar disorder. Specifically, we enriched for the PSD of the ACC in bipolar disorder and control samples and used mass spectrometry-based proteomic methods to characterize differential expression of the PSD-associated proteins. Overall, 288 proteins were found to be significantly differently expressed following FDR out of a total of 2033 protein identified and suitable for group comparison. The most notable proteomic changes involved those proteins with roles in mitochondrial functions and tricarboxylic acid cycle, oxidative phosphorylation and the processes of protein translation through EIF2 signaling, long-term potentiation, calcium signaling and endocytosis. All of these processes are important in synaptic plasticity. Together, these findings provide support for the view that abnormal protein expression within the PSD contributes to the pathophysiology of bipolar disorder through effects on synaptic plasticity.

The potential biological importance of the PSD to neuropsychiatric illness is high. The PSD is an electron-dense multiprotein complex under the postsynaptic membrane, which is readily identified by electron microscopy. It has been characterized previously in the rodent and human cortex using proteomic methods, and it contains many neuroreceptors such as n-methyl-d-aspartate, α-amino-3-hydroxy-5-methyl-4-isoxazolepropionic acid and metabotropic glutamate receptors, which influence long-term potentiation^51^ and synaptic plasticity, and which are implicated schizophrenia and bipolar disorder. In keeping with our current data, previous studies characterizing the PSD have shown that in addition to numerous neuroreceptors it contains many proteins associated with mitochondrial function, protein translation, long-term potentiation, calcium signaling and endocytosis. The PSD role in synaptic neurotransmission thus extends beyond structural components of receptor signaling into roles in which synaptic function is dependent such as energy metabolism^52^ and protein translation^23^ and calcium signaling.^53^ To our knowledge, no previous study has enriched for the PSD in bipolar disorder and assessed its differential protein expression compared with controls. Here, for we believe the first time, we demonstrate the importance of processes that underpin synaptic plasticity and mitochondrial functions in the bipolar disorder PSD proteome.

The findings reported here are relevant to the previous literature, implicating these processes in neuropsychiatric disorders, in particular schizophrenia. Our team recently applied this method to study the PSD in the ACC in schizophrenia and observed altered expression of proteins involved in endocytosis, long-term potentiation and calcium signaling.^25^ These findings are consistent with previous gene and protein expression studies of schizophrenia and, although to a lesser extent, bipolar disorder.^49^ Reduced expression of two proteins centrally involved in clathrin-mediated endocytosis, namely Dynamin1 and AP2, were described in the schizophrenia PSD. Interestingly, both these proteins were found to be dysregulated in the current study, arguing that the process of clathrin mediated endocytosis is disturbed in major psychiatric disorder. The processes of long-term potentiation and calcium signaling (see Table 2 and Supplementary Table 6) were also implicated in the PSD in both schizophrenia and the current study of bipolar disorder. This is in keeping with previous studies and a shared role of these processes in n-methyl-d-aspartate receptor function and synaptic plasticity.^51^ We previously observed altered expression of proteins with known roles in n-methyl-d-aspartate function, and in the current study we have observed significant downregulation of ARFGAP, PLP1, SHANK3, CAMK2B, SYNPO and PRDX1 and upregulation of AMPH. The mitochondrial-related proteins (ATP5H, SSBP1, SLC25A4 and NDUFV2) previously shown to be differentially expressed in the PSD in schizophrenia were also differentially expressed in the PSD in bipolar disorder.

Beyond our hypothesized findings, we observed highly significant upregulation for each Septin 3, 5, 7, 8, 9 and 11. Septins have roles in dendritic spine morphology,^54^ myelination,^55^ synaptic vescicle fusion,^56^ membrane curvature^57^ and cytoskeletal dynamics, which has been implicated in schizophrenia.^58^ Septin 5, 6 and 11 specifically have been implicated in schizophrenia and bipolar disorder previously in keeping with the proposed synaptic–dendritic basis to the disorders.^59^

It is informative to place the differentially expressed bipolar disorder proteome reported here with current genomic literature.

First, there is a 42% overlap between our study and a highly enriched functionally related PSD gene set reported in an integrative genomic study in bipolar disorder brain samples (17/40 proteins)^14^ (see Supplementary Table 7 for details of these findings).

Second, three of our differentially expressed proteins overlapped with the nine genes that were reported in a pathway meta-analysis of bipolar disorder and showed differential expression in the dorsolateral prefrontal cortex in bipolar disorder patients:^48^ inositol 1,4,5-trisphosphate receptor type 1 (*P*<0.005, overlap with ITPR2 isoform of 65% as determined by BLAST analysis), dystrobrevin alpha (DTNA, *P*<0.05) and neurotrophic tyrosine kinase receptor.

Third, Konradi *et al.*^29^ identified 43 genes altered at the mRNA level in the hippocampus in bipolar disorder patients. We have shown that 56% of those were also dysregulated in our differentially expressed bipolar disorder proteome reported here (see Supplementary Table 8 for details). Furthermore, the PSD is implicated with schizophrenia through several genome-wide association and whole-exome-sequencing studies. However, genome-wide association studies and pathway analysis of genome-wide association studies data from the Psychiatric Genomics Consortium have not identified PSD-associated genes in their primary findings as contributing to bipolar disorder risk. The lack of findings may be because of sample size differences between schizophrenia and bipolar disorder genome-wide association studies, and we wait with anticipation for the next installment from the Psychiatric Genetics Disorder on Bipolar Disorder, promising samples of >20 000 cases. Studies to date have implicated histone methylation,^21^ cAMP signaling,^22^ nuclear mitochondrial genes,^48^ although there is mounting evidence for mitochondrial DNA variants in schizophrenia,^60^ and to a lesser degree in bipolar disorder. More robust evidence, which would have an impact on mitochondrial function, is derived from genetic studies pointing to the involvement of the calcium channels in bipolar disorder, and studies of peripheral cells from subjects with bipolar disorder have shown alteration in calcium dynamics and energy production.^61^ Energy production is a function of mitochondria, and thus these changes are consistent with the current study.

The most robust finding in the current study is the upregulation of proteins with roles in mitochondrial function. While evidence from many studies implicates mitochondrial function in bipolar disorder, and indeed schizophrenia (for review see Konradi *et al.*^30^), the degree to which mitochondrial-associated proteins were dysregulated in the current study is more marked than that observed previously in the same Stanley series of brains (using unenriched material). This suggests that the mitochondrial-associated protein expression is most marked in the PSD fraction, a finding that may shed light on the findings of several transcriptomic studies, which did not demonstrate prominent alteration in mitochondrial-associated genes.^62^ Mitochondria are anatomically closely associated with synapses and are essential for the ATP generation, which in turn underpins the protein phosphorylation reactions that mediate synaptic signaling through calcium regulation, exocytosis, vesicle recruitment, potentiation of neurotransmitter release and synaptic plasticity.^23^ As the provision of an ATP supply power synapse-associated polyribosome complexes and clathrin-dependent endocytic machinery at postsynaptic sites, our observations of altered EIF2 signaling and endocytosis in the PSD in bipolar disorder could potentially be secondary to altered mitochondrial function. EIF2 signaling has been observed recently in two studies of schizophrenia-derived stem cells,^63, 64^ thus implicating the process of protein translation in both of the major psychosis. The findings raise the testable hypothesis that the major psychoses involve altered mitochondrial function at the level of the synapse that has an impact locally on protein translation and endocytosis, and so on altered synaptic plasticity. Altered trafficking patterns of mitochondria to postsynaptic densities may also contribute, and this possibility is supported by the observed altered expression in the current study of mitochondrial-trafficking proteins KIF1A, DynLL2 and the clathrin mediated endocytosis-associated protein Dynamin1. Further studies are warranted for a deeper understanding of the mitochondrial proteins, and to test whether the mitochondrial changes are indeed primary, and the synaptic changes secondary, as there also is the possibility that this could be the opposite.

Our study has several strengths and weaknesses. We used proteomic technology to identify differentially expressed proteins within the PSD in bipolar disorder. With more sensitive mass spectrometers being developed, the differences and the amount of identifications have been improved. Whereas our previous set of schizophrenia PSD samples were run on a linear ion trap (LTQ Orbitrap (at the end of 2012)), for the current study we used a quadrupole mass filter (Q-Exactive). We confirmed the protein expression changes of a number of proteins that were broadly representative of the main implicated functional pathways and processes using western blotting. We confirmed altered expression of proteins involved in clathrin mediated endocytosis, such as Dynamin1, Dystrobrevin (involved in the formation and stability of synapses), and the mitochondrial proteins NDUFV2 and SSBP; while NDUFV2 is involved in electron transfer, SSBP1 is involved in mitochondrial biogenesis and also confirmed Septin 11.

A further strength of our findings is the consistency of the direction of changes of proteins involved in several of the implicated pathways and processes. For example, 38 of the 39 mitochondria-associated proteins as shown by Ingenuity Pathway Analysis to be dysregulated in BPD were increased.

Postmortem studies have limitations that include the well-known confounds such as chronic exposure to neuroleptics, alcohol, tissue pH and postmortem delay that can confound these studies. We designed our study so that age, brain pH and sex were matched closely across groups. We could not account for the potential influence of medication effects, alcohol use, smoking or other recreational substance use, and future work will be needed to address these issues. In particular, it is known that antidepressants and smoking have effect on mitochondrial functions;^50, 65^ however, the direction of these influences is inconclusive.^66, 67^

In order to account for the potential effect of chronic exposure to neuroleptics in our bipolar disorder cases, we studied the PSD proteome of rats chronically exposed to haloperidol. Few changes were identified following this treatment, although four proteins—FAM163A, RIN1, mTOR and MCCC1—shown to be dysregulated in bipolar disorder were dysregulated in the same direction by haloperidol treatment, suggesting that these effects may have been drug treatment-related (see Supplementary Table 9). We also compared our findings with data from recently published articles of chronic treatment of rats with lithium or valproate.^68, 69, 70^ Nanavati *et al.*^69^ used proteomic methods to assess the PSD-enriched hippocampus following chronic treatment with lithium or valproate. Overall, there was little overlap with our findings, although they did observe dysregulation of ANK3, IGSF8 and CAPZB in the same direction as observed by us, suggesting that their findings may be drug treatment-related. Two other proteomic studies of the rat prefrontal cortex following chronic lithium or valproate treatment identified CNP, GAP43, MAP2, MAPRE2 and TCEB2 in the same direction as in the bipolar data set, indicating that these findings may be drug treatment-related (see Supplementary Table 9). However, we cannot fully confirm or exclude correlations within the patient group between the key proteins and medication as for this we would have to look at drug-naive patient samples that were not available to us. Previous studies are supportive of the view that mitochondrial hypofunction may be induced by antipsychotic agents, although dendritic morphology^71^ and mitochondrial gene expression^27^ are reported to be unchanged or largely reduced^72^ and mood stabilizers are considered to have a neuroprotective effect on mitochondria.^30^ It should also be appreciated that our understanding of the broader protein content of the PSD is based on proteomic studies of enriched samples and that the methods used can vary, leading to different PSD proteomes. The method used is seen as enrichment rather than a fractionation technique, and there is a possibility that the mitochondrial proteins in the PSD-enriched fraction might come from other cellular locations.

However, our PSD enrichment method is well published, and this along with our sensitive LC-MS/MS method yielded a PSD proteome, which is very similar to that described previously.

In conclusion, in this study, which is to our knowledge the first of its kind, we have identified and compared the unique set of proteins involved in the PSD in bipolar disorder. Our study provides unique insights into the disorder at the synaptic level and suggests that mechanisms involving altered mitochondrial function, protein translation, calcium signaling, endocytosis and septin function at the level of the PSD are involved in the pathophysiology of bipolar disorder. As these processes act in concert to support synaptic plasticity, they provide insights into the variety of processes that may be targeted in the search for novel treatments for the disorder.

## Figures and Tables

**Table 1:** Demographic information for the 36 samples used for analyzing protein expression by LC-MS/MS in the anterior cingulate cortex

	*Control*	*Bipolar disorder*
Number	20	16
Race	20 White	15 White 1 Black
Sex	15 M, 5 F	10 M, 6 F
Side of brain	11 Right, 9 left	8 Right, 8 left
Psychotic features	No	9 Yes, 5 no, 2 unclear
Cause of death	18 Cardiac 2 Other medical	4 Cardiac 7 Other medical 5 Suicide
Mean age and range (y)	43.6 (31–57)	46.6 (19–64)
Mean PMI and range (h)	21 (9–31)	25.8 (12–38)
Mean brain pH and range	6.59 (6.0–6.94)	6.46 (5.97–6.97)
Mean RI and range (h)	2.9 (0–7)	4.9 (1–10)^a^
Mean lifetime alcohol abuse (1=low, 5=high)	0.55 (0–3)	2.87 (0–5)
Smoking at time of death	3 Yes	7 Yes
	5 No	3 No
	12 Unknown	6 Unknown
Mean lifetime drug abuse (1=low, 5=high)	0.2 (0–1)	2.06 (0–5)
		
*Prescribed psychotropic medication use at time of death, no. of on/no. of off*		
Antipsychotics	0/20	6/10
Antidepressants	0/20	7/9
Mood stabilizers	0/20	11/5

Abbreviations: F, female; LC-MS/MS, liquid chromatography–mass spectrometry; M, male; PMI, postmortem interval; RI, refrigerator interval; y, years.

For alcohol and drug use, a categorical scale was employed: 0=little or none, 1=social use, 2=moderate past use, 3=moderate present use, 4=heavy past use and 5=heavy present use.

a Significantly different (*P*<0.05) from controls following independent *t*-test (disease versus control).

**Table 2:** Top five significant canonical pathways identified among the 288 differentially expressed proteins (*t*-test; *P*<0.007) between bipolar disorder and control samples

*Canonical pathway*	*Proteins*	P*-value*	*Ratio*
Oxidative phosphorylation	ATP5A1, ATP5B, ATP5C1, ATP5F1, ATP5H, ATP5J2, ATP5O, CYC1, CYCS, MT-ND4, MT-ND5, NDUFA2, NDUFA5, NDUFA6, NDUFA7, NDUFA8, NDUFA9, NDUFA10, NDUFA12, NDUFA13, NDUFB1, NDUFB3, NDUFB4, NDUFB9, NDUFB11, NDUFS1, NDUFS2, NDUFS3, NDUFS5, NDUFS6, NDUFV1, NDUFV2, UQCR10, UQCRC2, UQCRH, UQCRQ	1.06E−39	36/109 (0.33)
Mitochondrial dysfunction	ATP5A1, ATP5B, ATP5C1, ATP5F1, ATP5H, ATP5J2, ATP5O, CYC1, CYCS, GPD2, GPX4, MT-ND4, MT-ND5, NDUFA2, NDUFA5, NDUFA6, NDUFA7, NDUFA8, NDUFA9, NDUFA10, NDUFA12, NDUFA13, NDUFB1, NDUFB3, NDUFB4, NDUFB9, NDUFB11, NDUFS1, NDUFS2, NDUFS3, NDUFS5, NDUFS6, NDUFV1, NDUFV2, PDHA1, PRDX3, UQCR10, UQCRC2, UQCRH, UQCRQ	2.57E−37	40/171 (0.234)
EIF2 signaling	EIF2B3, EIF2S3, EIF3C, EIF4G2, PDPK1, PPP1CB, PPP1CC, RPL3, RPL6, RPL7, RPL8, RPL9, RPL12, RPL18, RPL13A, RPS2, RPS5, RPS10	1.62E−10	18/185 (0.097)
Calcium signaling	ATP2A2, ATP2B2, CAMK2B, CAMKK1, ITPR1 LETM1, PPP3CA, PPP3R1, PRKACB, PRKAR2B, RYR2, SLC8A2, TPM1, TPM3	2.64E−07	14/178 (0.079)
TCA cycle	CS, DLD, DLST, IDH3B, IDH3G, MDH2	6.36E−07	6/23 (0.26)

Pathways were identified using Ingenuity Pathway Analysis (Qiagen, Redwood City, CA, USA). Significant pathways are determined using the Fisher's exact test to compare the number of proteins that are most significantly perturbed in the data.

**Table 3:** Summary of validation work

*Protein Id*	*Protein name*	*LC-MS/MS*		*Validation (western blots)*		*Rodent western blots (*P*-value)*
		*Direction*	P*-value*	*Direction*	P*-value*	
1740	DNM1	−	0.0006	−	0.039	0.731
787	DTNA	−	0.013	−	0.029	0.86
319	NDUFV2	+	0.0037	+	5.6 × 10^−6^	0.55
282	SEPT11	+	0.0004	+	2.46 × 10^−7^	0.715
347	SSBP	−	0.0006	+	0.019	0.703
2415	GAD67	−	0.03	−	0.1	0.714

Abbreviations: LC-MS/MS, liquid chromatography–mass spectrometry; PSD, postsynaptic density.

Validation was undertaken using western blot analysis for DNM1, DTNA, NDUFV2, SEPT11 and SSBP1 in the PSD-enriched samples from the Stanley Foundation Array series. In keeping with our LC-MS/MS experiments, DNM1 and DTNA1 expression was reduced, and SEPT11 and NDUF2 were found to be increased. SSBP was found to be decreased in the MS data but increased by western blotting. This most probably is due to the antibody recognizing different/all isoforms of the protein. Western blot analyses demonstrated that DNM1, DTNA, NDUFV2, SEPT11 and SSBP1 were not significantly altered in rats treated with haloperidol.
