# Supplementary material for: Proteomic analysis of the postsynaptic density implicates synaptic function and energy pathways in bipolar disorder
Source: Transl Psychiatry. 2016 Nov 29;6(11):e959–. doi: 10.1038/tp.2016.224 (PMC5290351; doi:10.1038/tp.2016.224)
Supplement: Supplementary Methods [file tp2016224x1.doc]

**Supplementary Methods**

**PSD enrichment in ACC in bipolar disorder and controls**

Five-hundred mg of brain tissue was homogenized in 2.5 ml of solution A (0.1 mM CaCl_2_, 1 mM MgCl_2_ and 0.32 M sucrose). This homogenate was adjusted to 1.25 M Sucrose and 0.1 mM CaCl_2_ by adding 3.35 ml 2M Sucrose and 0.15 ml 0.1 M CaCl2. 5 ml of 1 M sucrose was overlaid on this and ultracentrifuged at 28,000 rpm (100,000×g) for 4 hrs up to overnight in a SW 40 Ti rotor using a Beckmann L7 Ultracentrifuge.

The band at the interface of 1.25 and 1 M sucrose was collected with a needle as the synaptic membrane fraction (SMF).

The SMF was diluted with 5× 0.1 mM CaCl_2_ and centrifuged at 12000 rpm (15000×g) for 20 minutes. The pellet was solubilized in 600 µl 20 mM Tris pH 7.4 and sonicated with three 10 second pulses and the samples were left for end over end shaking for 60 min in 4°C. For the PSD preparation, 500 µl of SPM was diluted with ice cold 0.1 mM CaCl_2_ (2ml) to 2.5 ml, followed by the addition of 40 mM Tris-HCl, pH 6 supplemented with 2% Triton-X 100, bringing the final volume to 5 ml. The samples were left in cold room rocker for 30 minutes and centrifuged at 18,000 rpm (35,000×g) for 20 minutes. The supernatant, designated as the synaptic vesicle fraction, was then diluted with 5× chilled acetone (25 ml) and left in −20°C overnight. The pellet from the last round of centrifugation was air dried and dissolved in 1 ml 0.1 mM CaCl_2_, then to this 1 ml 40 mM Tris pH 8 with 2% Triton-X 100, was added to the final concentration of 20 mM Tris and 1% Triton-X. The sample was left on a rocker for 60 minutes and centrifuged at 36,000 rpm (140,000×g) for 30 minutes. The supernatant, designated as the presynaptic membrane fraction was acetone precipitated with 5 x chilled acetone (10 ml) and left at −20°C overnight. The pelleted PSD was air dried and dissolved in 70 µl 20 mM Tris pH 7.4 with sonication and 1 hour of rocking. Acetone precipitated fractions were centrifuged at 15,000 rpm (24,000×g) for 30 minutes supernatants discarded and pellets were dissolved in 70 µl for the synaptic vesicle fraction and 50 µl for presynaptic membrane and postsynaptic density in 20 mM Tris pH 7.4 by sonication and 30 min rocking in the cold room.

The purity of PSD fractions was determined by the extent to which they contained presynaptic protein and synaptic membrane proteins. Samples were tested for PSD-95 and synaptophysin using immunoblot analysis of fractions (as described below).

**Classification of findings**

DAVID NIH was used for pathway analysis according to the Kyoto Encyclopedia of Genes and Genomes (KEGG^TM^; <http://david.abcc.ncifcrf.gov/>) using two lists, 1) the total PSD protein list identified by us (n=2033), and 2) the PSD proteins differentially expressed between bipolar disorder and control (n=620, p<0.05; n=288 significant at 5% FDR).

The String (Search Tool for the Retrieval of Interacting Genes) database[^1^](#_ENREF_1) (http://string-db.org) was used to visualize functional protein association networks.

Ingenuity Pathway Analysis (IPA®, QIAGEN Redwood City, www.qiagen.com/ingenuity) was used to analyze the key biological relationships of all differentially expressed proteins. Significant biological functions are determined using Fisher exact test to compare the number of proteins that are most significantly perturbed in the data set.

**Validation of differentially expressed proteins**

***Human Tissue***

We selected proteins for validation based on their degree of differential expression, the rank of the fold change differences in expression and biological relevance. As our hypothesis focused on overlap with schizophrenia, we also targeted these proteins where antibodies were available and working. Validation was undertaken using Western blotting for DNM1, DTNA, NDUFV2, SEPT11, and SSBP1 in the same samples (10 control and 8 BD sub-pools) from the Stanley Foundation Array series. Neither ELISA nor Western Blots were successfully optimized for four of the proteins chosen, namely SHANK3, SLC25A, RPH3A and MAPRE2.

***Western blotting***

Protein concentration was measured by the Bradford method[^2^](#_ENREF_2). Equal concentrations of denatured PSD protein (10 μg/lane) were loaded and resolved on 12% SDS-polyacrylamide gels, the proteins were separated by electrophoresis and transferred onto nitrocellulose membranes. The specificity of primary antibodies used for validation experiments was shown by western blot in human test tissue. Each protein migrated with a single band of the predicted molecular weight, PSD-95 (95 kDa, Millipore, 1:5000), synaptophysin (38 kDa, DAKO, 1:5000), Dynamin1 (DNM1, 120 kDa, Abcam, 1:20,000), Dystrobrevin (DTNA, 84 kDa, Abcam, 1:5000), NADH dehydrogenase (ubiquinone) flavoprotein 2 (NDUFV2, 24 kDa, Abcam, 1:10000), Septin 11 (SEPT11, a gift from Dr Koh-ichi Nagata[^3^](#_ENREF_3)), and single-stranded DNA binding protein 1 (SSBP1, 17 kDa, Abcam, 1:1000) were used as primary antibodies. The corresponding horseradish peroxidase-conjugated secondary antibodies (anti-rabbit IgG (1:2000-1:4000 dilution, Promega) and anti-mouse IgG (1:2000 dilution, Promega)) were used. Blots were incubated with ECL chemiluminescent reagent (Amersham Biosciences), exposed and digitalized with the ChemiDoc Imaging System (BioRad Laboratories). Levels of expression were quantified and normalized against anti-(rabbit) ERK2 (1:8000, Santa Cruz Biotechnology) using ImageLab software (BioRad Laboratories).

***Haloperidol-treated rats***

To assess the effects of antipsychotic medication on the expression of candidate proteins, cortex tissue harvested from rats chronically treated with haloperidol[^4^](#_ENREF_4) and enriched for the PSD previously by us as well as the data from the previous mass spectrometry analysis was used[^5^](#_ENREF_5). Western blotting was undertaken on rat cortex PSD fractions using primary antibodies to DNM1, DTNA, NDUFV2, SEPT11, and SSBP1 to assess differential expression of protein as described above.

**Statistical Analysis of validation work**

For western blot analyses, density values were measured and corrected by the signal intensity of the respective antibody to ERK2. An independent sample t-test was used to analyze differences in the groups of interest with statistical significance set at the 5% level. A minimum of at least three experiments were performed for each protein of interest.

1. Szklarczyk D, Franceschini A, Kuhn M, Simonovic M, Roth A, Minguez P *et al.* The STRING database in 2011: functional interaction networks of proteins, globally integrated and scored. *Nucleic Acids Res* 2011; **39**(Database issue)**:** D561-568.

2. Bradford MM. A rapid and sensitive method for the quantitation of microgram quantities of protein utilizing the principle of protein-dye binding. *Analytical biochemistry* 1976; **72:** 248-254.

3. Li X, Serwanski DR, Miralles CP, Nagata K, De Blas AL. Septin 11 is present in GABAergic synapses and plays a functional role in the cytoarchitecture of neurons and GABAergic synaptic connectivity. *J Biol Chem* 2009; **284**(25)**:** 17253-17265.

4. Hakansson K, Pozzi L, Usiello A, Haycock J, Borrelli E, Fisone G. Regulation of striatal tyrosine hydroxylase phosphorylation by acute and chronic haloperidol. *European Journal of Neuroscience* 2004; **20**(4)**:** 1108-1112.

5. Focking M, Lopez LM, English JA, Dicker P, Wolff A, Brindley E *et al.* Proteomic and genomic evidence implicates the postsynaptic density in schizophrenia. *Mol Psychiatry* 2015; **20**(4)**:** 424-432.
