# Supplementary figures and images for: Proteomic analysis of the postsynaptic density implicates synaptic function and energy pathways in bipolar disorder
Source: Transl Psychiatry. 2016 Nov 29;6(11):e959–. doi: 10.1038/tp.2016.224 (PMC5290351; doi:10.1038/tp.2016.224)

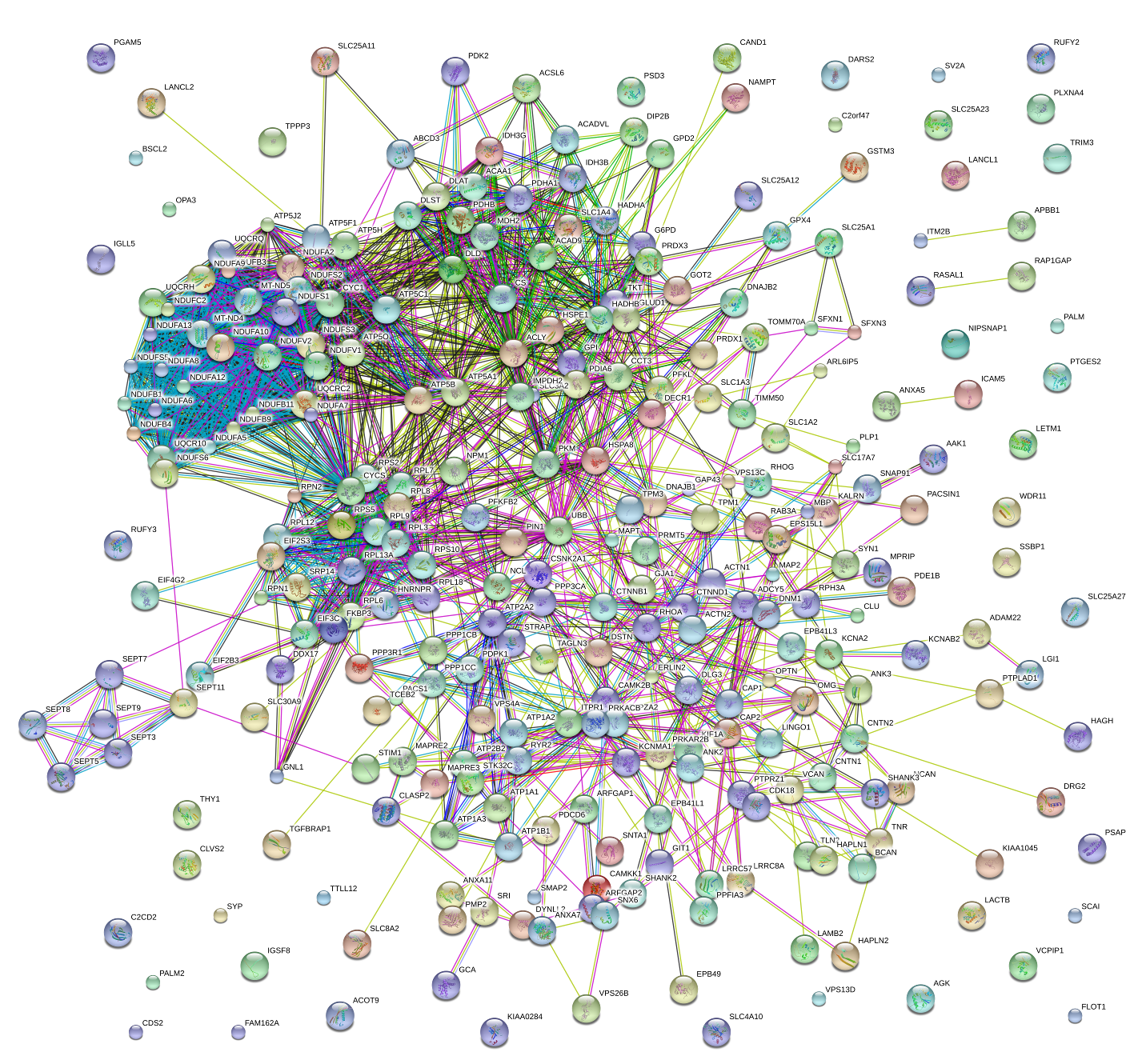

Supplement: Supplementary Figure 1 [file tp2016224x2.doc]
