# Supplementary material for: Proteomic analysis of the postsynaptic density implicates synaptic function and energy pathways in bipolar disorder
Source: Transl Psychiatry. 2016 Nov 29;6(11):e959–. doi: 10.1038/tp.2016.224 (PMC5290351; doi:10.1038/tp.2016.224)
Supplement: Supplementary Table 1 [file tp2016224x3.doc]

**Supplementary Table 1**

| **Control** | | | | |  | **Bipolar Disorder** | | | | |
| --- | --- | --- | --- | --- | --- | --- | --- | --- | --- | --- |
| **Age (y)** | **Sex** | **Brain pH** | **PMI (h)** | **RI (h)** |  | **Age (y)** | **Sex** | **Brain pH** | **PMI (h)** | **RI (h)** |
|  |  |  |  |  |  |  |  |  |  |  |
| 45 | M | 6.81 | 18 | 2 | 1 | 35 | M | 6.58 | 22 | 4 |
| 47 | M | 6.81 | 21 | 2 |  | 33 | F | 6.51 | 24 | 4 |
|  |  |  |  |  |  |  |  |  |  |  |
| 38 | F | 6.7 | 28 | 3 | 2 | 44 | M | 6.74 | 19 | 5 |
| 44 | F | 6.59 | 28 | 3 |  | 48 | M | 6.90 | 23 | 6 |
|  |  |  |  |  |  |  |  |  |  |  |
| 48 | M | 6.91 | 24 | 6 | 3 | 48 | F | 6.50 | 18 | 4 |
| 49 | M | 6.93 | 23 | 4 |  | 51 | M | 6.67 | 23 | 4 |
|  |  |  |  |  |  |  |  |  |  |  |
| 47 | M | 6.6 | 11 | 3 | 4 | 35 | M | 6.30 | 35 | 6 |
| 53 | M | 6.4 | 9 | 2 |  | 45 | M | 6.03 | 35 | 6 |
|  |  |  |  |  |  |  |  |  |  |  |
| 33 | F | 6.52 | 29 | 3 | 5 | 64 | M | 6.10 | 16 | 1 |
| 34 | M | 6.48 | 22 | 1 |  | 56 | M | 6.07 | 23 | 3 |
|  |  |  |  |  |  |  |  |  |  |  |
| 45 | M | 6.94 | 29 | 4 | 6 | 42 | M | 6.65 | 32 | 3 |
| 51 | M | 6.7 | 31 | 2 |  | 49 | F | 6.39 | 38 | 2 |
|  |  |  |  |  |  |  |  |  |  |  |
| 31 | M | 6.13 | 11 | 3 | 7 | 58 | F | 6.50 | 35 | 7 |
| 37 | M | 6.5 | 13 | 2 |  | 56 | F | 6.58 | 26 | 10 |
|  |  |  |  |  |  |  |  |  |  |  |
| 57 | M | 6.4 | 26 | 0 | 8 | 63 | F | 6.97 | 32 | 6 |
| 53 | M | 6 | 28 | 2 |  | 19 | M | 5.97 | 12 | 8 |
|  |  |  |  |  |  |  |  |  |  |  |
| 32 | M | 6.57 | 13 | 6 | 9 |  |  |  |  |  |
| 44 | F | 6.2 | 10 |  |  |  |  |  |  |  |
|  |  |  |  |  |  |  |  |  |  |  |
| 51 | M | 6.71 | 22 | 7 | 10 |  |  |  |  |  |
| 34 | F | 6.87 | 24 | 2 |  |  |  |  |  |  |
