# Supplementary material for: Proteomic analysis of the postsynaptic density implicates synaptic function and energy pathways in bipolar disorder
Source: Transl Psychiatry. 2016 Nov 29;6(11):e959–. doi: 10.1038/tp.2016.224 (PMC5290351; doi:10.1038/tp.2016.224)
Supplement: Supplementary Table 4 [file tp2016224x6.doc]

Supplementary Table 4.

| **Bipolar disorder**   | **Schizophrenia** |  | | --- | --- | | Oxidative phosphorylation | Oxidative phosphorylation | | Ribosome | Calcium signaling pathway | | Endocytosis | Regulation of actin cytoskeleton | | Regulation of actin cytoskeleton | Endocytosis | | Focal Adhesion | Gap junction | | Tight Junctions | Tight junction | | Calcium signaling pathway | Oocyte meiosis | | Insulin signaling pathway | Longterm potentiation | | Chemokine signaling pathway | Glycolysis / gluconeogenesis | | Gap junction | Pathogenic E. coli infection | |  |
| --- | --- | --- | --- | --- | --- | --- | --- | --- | --- | --- | --- | --- | --- | --- | --- | --- | --- | --- | --- | --- | --- | --- | --- |
|  |  |
|  |  |
|  |  |
|  |  |
|  |  |
|  |  |
|  |  |
|  |  |
|  |  |
|  |  |
