# Supplementary material for: Proteomic analysis of the postsynaptic density implicates synaptic function and energy pathways in bipolar disorder
Source: Transl Psychiatry. 2016 Nov 29;6(11):e959–. doi: 10.1038/tp.2016.224 (PMC5290351; doi:10.1038/tp.2016.224)
Supplement: Supplementary Table 6 [file tp2016224x8.doc]

| **Canonical Pathway** | **p-value** | **ratio** |
| --- | --- | --- |
| ***Mitochondrial Dysfunction*** | 1.71 E-47 | | 60/171 (0.351) | | --- | |
| ***Oxidative Phosphorylation*** | 1.07E-46 | 50/109 (0.459) |
| ***EIF2 Signaling*** | 8.22E-15 | 31/185 (0.168) |
| ***Remodeling of Epithelial Adherens Junctions*** | 1.32E-11 | 17/68 (0.250) |
| ***Synaptic Long-Term Potentiation*** | 4.69E-10 | 20/1191 (0.168) |

Supplementary Table 6.
